# Behavioral regression in shank3^Δex4−22^ mice during early adulthood corresponds to cerebellar granule cell glutamatergic synaptic changes

**DOI:** 10.21203/rs.3.rs-4888950/v1

**Published:** 2024-09-06

**Authors:** Rajaram Kshetri, James O Beavers, Romana Hyde, Roseline Ewa, Amber Schwertman, Sarahi Porcayo, Ben D Richardson

**Affiliations:** 1 Department of Pharmacology, Southern Illinois University – School of Medicine, Springfield, IL 62702; 2 Department of Biological Sciences, University of Idaho, Moscow, ID 83844

**Keywords:** Shank3, autism spectrum disorder, Phelan-McDermid syndrome, regression, cerebellum, glutamate receptor, AMPAR, granule cell, mouse behavior phenotype

## Abstract

**Background:**

*Shank3*, a gene encoding a synaptic scaffolding protein, is implicated in autism spectrum disorder (ASD) and is disrupted in Phelan-McDermid syndrome (PMS). Despite evidence of regression or worsening of ASD-like symptoms in individuals with PMS, the underlying mechanisms remain unclear. Although *shank3* is highly expressed in the cerebellar cortical granule cells, its role in cerebellar function and contribution to behavioral deficits in ASD models are unknown. This study investigates behavioral changes and cerebellar synaptic alterations in *shank3*^*Δex4−22*^ mice at two developmental stages.

**Methods:**

S*hank3*^*Δex4−22*^ wildtype, heterozygous, and homozygous knockout mice lacking exons 4–22 (all functional isoforms) were subjected to a behavioral battery in both juvenile (5–7 weeks old) and adult (3–5 months old) mouse cohorts of both sexes. Immunostaining was used to show the expression of SHANK3 in the cerebellar cortex. Spontaneous excitatory postsynaptic currents (sEPSCs) from cerebellar granule cells (CGCs) were recorded by whole-cell patch-clamp electrophysiology.

**Results:**

Deletion of *shank3*^*ex4−22*^ caused deficits in motor function, heightened anxiety, and repetitive behaviors. These genotype-dependent behavioral alterations were more prominent in adult mice than in juveniles. Reduced social preference was only identified in adult *shank3*^*Δex4−22*^ knockout mice and self-grooming was uniquely elevated only in males across both age groups. Immunofluorescence staining indicates the presence of SHANK3 predominantly in the dendrite-containing rosette-like structures in CGCs, colocalizing with presynaptic markers of glutamatergic mossy fiber. Electrophysiological findings identify a parallel relationship between the age-related exacerbation of behavioral impairments and the enhancement of sEPSC amplitude in CGCs.

**Limitations:**

Other behavioral tests of muscle strength (grip strength test), memory (Barnes/water maze), and communication (ultrasonic vocalization), were not performed. Further study is necessary to elucidate how SHANK3 modulates synaptic function at the mossy fiber-granule cell synapse in the cerebellum.

**Conclusions:**

Our findings reveal an age-related exacerbation of behavioral impairments in *shank3*^*Δex4−22*^ mutant mice. These results suggest that SHANK3 may play a role in maintaining glutamatergic receptors and synapses in CGCs, as well as the potential involvement of the cerebellum in ASD.

## Background

It is estimated that one in every 36 children in the United States is diagnosed with autism spectrum disorder (ASD) [1]. Although the precise underlying causes and neurological mechanisms of ASD are poorly understood and likely diverse, disruption of multiple genes is linked to ASD [2–7]. Of the genes most strongly associated with ASD in recent genome-wide association studies [6–8], the *shank3* gene is consistently identified and has long been considered a potential monogenic cause of ASD [9,10]. Haploinsufficiency of *shank3*, arising from mutations, deletions [4,11–13], or epigenetic modifications [14] that disrupt SHANK3 protein expression or function is identified in a notable proportion (0.5–2%) of individuals with autism spectrum disorder (ASD) and is the primary cause of Phelan-McDermid syndrome (PMS, 22*q*13.3 deletion) [15–18]. PMS is characterized by a high prevalence of syndromic ASD (84%) or intellectual disability (77%) [11,19]. Although clinical data suggest that the majority of individuals with altered *shank3* expression (i.e. PMS) undergo a delayed regression or worsening of ASD-like behaviors [20–25], the timing and extent to which animal models with *shank3* mutations/deletions recapitulate this regression is still emerging [26–29].

*Shank* genes (*shank1*, *2*, and *3*) encode a family of multi-domain-containing proteins that serve as synaptic scaffolding and regulatory proteins for NDMA, AMPA, and metabotropic (mGluR) glutamate receptors at postsynaptic densities [13,30–32]. Due to splice variants of its 22 exons, the SHANK3 protein has six isoforms (A-F) that are uniquely expressed in particular brain regions [13,33], with mouse behavioral phenotype and changes in neuronal function varying based on the *shank3*/SHANK3 exons/isoforms deleted [13,34–47]. Unfortunately, the deletion of *shank3* isoforms from specific cell types or brain areas in rodents, like forebrain and striatum [41] has not yet led to a clear understanding of where in the brain *shank3*/SHANK3 is critical for shaping all behavioral domains affected by *shank3* disruption.

One area of the brain in which *shank3*/SHANK3 expression steadily increases [33] during development and through adulthood is the cerebellar cortex, particularly SHANK3C/D isoforms in cerebellar granule cells (CGCs) [33,44–46,48], where glutamate receptor (AMPAR, NMDAR, mGluR) function is important for both development [49,50] and synaptic processing by mature CGCs [51–56]. Despite the role of SHANK3 in the regulation of AMPARs, NMDARs, and mGluRs and its expression in developing and adult CGCs, only one study has evaluated the role of *shank3*/SHANK3 in the cerebellum, identifying deficits in cerebellar learning in heterozygous *shank3*^*Δex21*^ mice [40]. Given the established link between cerebellar dysfunction and ASD [10,57–64] and the high level expression of ASD-linked genes in the cerebellum [7,65], understanding the role of SHANK3C/D isoforms in even basal synaptic function of CGCs may be an important component of conceptualizing PMS and ASD.

Although the cerebellum is well-described for its role in motor control and motor learning [66,67], the crystalline-like cerebellar cortex shapes activity of the cerebellum afferents that project to motor and many non-motor brain areas as well [68–71]. These mono- and polysynaptic connections to non-motor brain areas are diverse (e.g. hypothalamus, ventral tegmental area, hippocampus) and, along with functional studies, indicate cerebellar involvement in cognitive, affective, reward, motivation, and sensory processing [60,68,72–79]. This expansion of brain areas and functions that involve the cerebellum thereby establishes a rich network of interactions by which cerebellar dysfunction may impact a broad array of neural functions, processes, and behaviors.

Given that most individuals with PMS or disruption of the *shank3* gene undergo behavioral regression during childhood and adolescence that continues into early adulthood [20–25], leveraging an ideal animal model displaying similar regression may be key to identifying brain regional and molecular mechanisms that drive this regression. Preferred assessment of animal model behavior should account for wildtype/heterozygous/homozygous genotypes, both sexes, age ranges analogous to key human age ranges, circadian effects (light vs. dark phase), and behavior across a range of domains. Accounting for these factors and to bridge gaps in current literature assessing behavioral regression in the absence of some or all SHANK3 isoforms [26–29], we assessed the behavioral phenotype of male and female *shank3*^*Δex4−22*^ mice in two separate age cohorts during the dark phase. Then, to determine whether cerebellar dysfunction corresponds to the development of the behavioral phenotype across early adulthood, we evaluated the expression of SHANK3 at CGC synapses and differences in spontaneous excitatory postsynaptic currents in wildtype, heterozygous, and knockout *shank3*^*Δex4−22*^ mice corresponding to both age cohorts and sexes.

## Materials & Methods

### Animals

All procedures involving animals were performed in accordance with protocols approved by the Institutional Animal Care and Use Committee at Southern Illinois University – School of Medicine or the University of Idaho. *Shank3*^*Δex4−22*^ (JAX stock no.: 032169) and C57bl/6J mice (JAX stock no.: 000664) were initially acquired from Jackson Laboratories and/or bred in-house to generate animals used in all experiments. *Shank3*^*Δex4−22*^ mice were maintained on a C57BL/6NJ genetic background as provided by the vendor [42,47]. These mice lacking exons 4–22 of the *shank3* gene lack expression of all major isoforms A-F that are differentially expressed throughout the brain [33,42,47]. A heterozygous/heterozygous breeding strategy was employed to generate s*hank3*^*Δex4−22*^ wildtype (+/+, WT), heterozygous (−/+, Het), and homozygous knockout (−/−, KO) mice used for all behavioral and electrophysiology experiments. Offspring genotypes were determined through Transnetyx (Cordova, TN) using ear punch or tail biopsies. All mice were group housed with one to three other mice on a reversed (12hr/12hr) light-dark cycle with *ad libitum* access to food and water.

### Behavioral Battery

Mice of both sexes representing all three s*hank3*^*Δex4−22*^ genotypes were randomly tested on a behavioral battery to assess motor, anxiety, sociability, repetitive, and memory behaviors described below with the number of assays and order of completion for each cohort chosen randomly. Some mice did not always complete all assays. Separate cohorts of mice were evaluated on the behavioral battery at either a juvenile (5–7 weeks) or young adult age (3–4 months) with no mice exposed to the same assay more than once in their lifetime. All behavioral testing was completed in low red-light conditions (15–20 lux) during the dark phase. Video tracking and automated analysis (Noldus EthovisionXT v17.5) of animal behavior in open field, elevated zero maze, Y-maze, and sociability assays were used to evaluate animal location. For all other assays and manual scoring in an open field, experimenters were blinded to the genotype of all mice during testing and for manual analysis. Prior to each behavioral test, animals were habituated for 30 minutes in the testing room and each apparatus was thoroughly cleaned to reduce the impact of odor cues that may interfere with behavior.

### Open Field

Mice were evaluated in an open field (40 × 40 cm box) for a total of 30 minutes to assess gross motor function, locomotor behavior, and other stereotypical behavioral patterns. Mice were observed in the open field to determine total voluntary distance traveled, time within the center (20 × 20 cm) region equidistant from all edges, total entries into the center region, freezing time, total amount of time spent grooming, maximal speed, and total number of fecal boli deposited. To determine mouse location within the arena, the center point of the body was used.

### Elevated Zero Maze

The elevated zero maze comprises a circular (5 cm wide) track with an inner diameter of 40 cm that is elevated 60 cm above the ground. The annulus is divided into four equal quadrants, wherein two opposing quadrants are left open and the remaining two alternate quadrants are enclosed by 40cm high opaque walls. The mice were placed in an open arm and allowed to freely explore the maze with various parameters, such as the duration spent in the open quadrants and the number of entries into the open quadrants were assessed. Mice that spend more time in the closed quadrants and exhibit fewer entries into the open quadrants are generally considered to have higher levels of anxiety, and vice versa. To determine mouse location within the arena, the center point of the body was used.

### Rotarod

To evaluate motor and vestibular function, mice were evaluated to determine the duration of time they were able to remain on a rotating rod (3.17 cm diameter, IITC Life Sciences, Inc., Woodland Hills, CA, USA) that was continuously accelerating from 4 – 40 RPM over a 5 minute period. Each mouse was tested on the rotarod for three trials per day for two consecutive days (six total trials), with a 10 minute intertrial interval. For each trial, the time at which a mouse remained attached to the rotating rod for one complete rotation and the time at which the mouse fell from the rod to the landing platform was recorded. A reduced latency to fall will indicate motor deficits, and a lack of improvement in subsequent trials indicates reduced motor learning ability [46,47].

### Beam Balance

To evaluate fine motor coordination and balance that might not be detected by other tests of more gross motor function, mice were placed at the one end of a horizontal flat beam (1 m long, 12 mm or 6 mm wide) and allowed to walk across the beam to a dark goal box (20 cm cube). First, mice were trained for two consecutive days, consisting of three trials on both the 12 mm and 6 mm beam with each trial for a given beam separated by a 1 minute rest period in addition to a 10 minute rest period between each beam. Subsequently, each mouse’s performance was evaluated on both beams on the third day when were tested on each beam twice. Performance during the test day was analyzed to determine the time to reach the dark box and the number of paw slips while traversing the beam [47,80]. Beam crossing time and total number of foot slips are an average of the two test trials.

### Gait Analysis

Footprint analysis was used to quantify potential variations in gait as an indicator of fine motor functional capacity. Mice were first trained to traverse a corridor runway (1 m long × 5 cm wide) lined with white standard electrocardiograph paper with a dark goal box placed at the opposite end of the corridor. After three training trials, the mouse’s paws were coated with nontoxic blue (front paws) or red (hind paws) paint to record paw placement on two consecutive runs. Stride length and width of the forelimbs and hindlimbs were determined by measuring the respective distances from the paw center as shown in Fig. 4A for the second test trial [80,81].

### Marble Burying

Burying of small objects is a naturalistic behavior in mice with changes in the engagement in this behavior proposed to be related to anxiety-like, repetitive, compulsive, and/or perseverative behavior [82,83]. First, each mouse was placed in an empty clean standard mouse cage (17 × 28 × 13 cm) with 3 cm of bedding for 5 minutes. Then, the mouse was removed and sixteen marbles were placed in the cage on top of the bedding. The mouse was then placed back in the cage and their activity recorded for 30 min. The video record was evaluated to determine the number of marbles that are at least 50% covered by bedding at each 5 min time interval.

### Y-Maze Spatial Working Memory

To assess short term working memory, mice were placed in the center of a “Y”-shaped maze composed of three 35 cm long arms (5 cm wide) extending out from a central point at 120° from one another with 20 cm tall walls. Mice were allowed to freely explore the novel Y-maze environment for 10 minutes with the center point of the mouse’s body crossing into the arm considered as an entry. The total number of arm entries recorded to assess exploratory behavior and the percentage of alternate arm entries into the least recently visited arm (as opposed to the most recently visited arm) was taken as a measure of short-term working memory function [84].

### Three-Chamber Sociability

To evaluate social behavior, mice were evaluated in a four-phase protocol within an arena (40.5cm wide, 60cm long, and 22cm high) that was divided into three equal-sized chambers with openings in the dividers to allow mice to travel move into each chamber. The center chamber of the arena was empty and the two chambers at opposing ends each contained one circular barred cage in the center of the chamber. The sociability assay protocol consisted of four 5 minute-long phases with the mouse placed back into the center chamber with doors between each chamber closed in between each phase. First, the test mouse was placed in the center chamber of the apparatus with the two empty cages present and the mouse was allowed to freely explore all three chambers. In the second phase pre-test phase, each mouse was allowed to explore the entire arena with two identical inanimate objects inside each cage. In the third phase, the mouse was given the opportunity to freely explore the arena with one of the inanimate objects replaced with an unfamiliar wildtype mouse of a similar age and same sex and a novel non-social stimulus (inanimate object) contained within the other cage. Finally, in phase four, the non-social inanimate object was replaced with another unfamiliar wildtype mouse of a similar age and same sex to serve as a novel social stimulus, then the test mouse was allowed to interact with both familiar and unfamiliar mouse. The amount of time the mouse spent within 2 cm of the cage containing the social stimulus (T_S_), non-social stimulus (T_NS_), familiar mouse (T_F_) and novel mouse (T_N_) were quantified and used to calculate the social preference index (I_SP_ = (T_S_ − T_NS_)/(T_S_ + T_NS_)) or social novelty index (I_SN_ = (T_N_ – T_F_)/(T_N_ + T_F_)) [85]. During the sociability assay, we observed that eight mice (2 WT, 6 KO) displayed a strong bias toward one side of the chamber that never entered one side of the sociability chamber. This complete absence of time spent in one side of the chamber made index calculations problematic and were therefore not included in analysis of social preference and social novelty preference behavioral data.

### Immunohistochemistry

For immunohistochemical analysis of SHANK3 distribution in the cerebellar cortex, male C57BI/6J mice were anesthetized using isoflurane (3–5%) and then transcardially perfused with 1X phosphate buffered saline (PBS) followed by 4% formaldehyde diluted in 1X PBS. Brains were then removed and post-fixed for 48–72 hours in 4% formaldehyde followed by placement into 30% sucrose in 1X PBS for at least 24 hours prior to sectioning. Sagittal 40μm thick slices of the cerebellum were prepared on a cryostat. Sagittal sections of the cerebellar vermis were then washed in 1X PBS and then permeabilized and blocked in 95% methanol 5% acetic acid for 10 min followed IHC/ICC Blocking Buffer (eBiosciences) with 0.5% triton-X 100 for 1 hr. To block endogenous IgG and reduce labeling by mouse primary antibodies, all slices were subject to a second blocking step of polyclonal goat F(ab) anti-mouse IgG (1:100; ab6668, Abcam) diluted in 1X PBS. Tissue sections were then incubated at room temperature for 4 hrs in primary antibodies that included monoclonal mouse IgG1 anti-VGlut1 (1:500; Neuromab/Ab Inc., #75–066), polyclonal chicken IgG anti-VGlut2 (1:500; Synaptic Systems, #135–416), and polyclonal rabbit anti-SHANK3 (1:1000; Alomone, APZ-013). Primary antibodies were then labeled for 2 hrs at room temperature with secondary antibodies conjugated to fluorescent tags diluted with 1X PBS that included goat anti-chicken AlexaFluor488 (1:500; Invitrogen, A11039), goat anti-mouse IgG1 AlexaFluor568 (1:500; Invitrogen, A21124), and donkey anti-rabbit AlexaFluor647 (1:500; Invitrogen, A31573). Immediately after immunolabeling, tissue sections were washed and transferred to glass slides and mounted with Prolong Gold (Invitrogen). Multi-plane confocal images were acquired using 4x, 20X, and 60X objective magnification with comparable image settings on a Nikon Spinning Disk Confocal Microscope.

To determine the degree of colocalization postsynaptic SHANK3 in cerebellar granule cell (CGC) dendrites with presynaptic VGlut1- and VGlut2-positive mossy fibers (MFs), multi-color single plane confocal images were evaluated using the Mander’s Coefficient. Ranging from 0 to 1, the Mander’s Coefficient indicates the proportion of the colocalizing pixels in each color channel, which is less sensitive to background noise than Pearson’s R [86]. Specifically, the Mander’s Coefficient using the auto-threshold regression of the target channel was used to assess colocalization within manually selected regions of interest (ROI) based on the profile of presynaptic MF (VGlut1 or VGlut2) terminals (see Fig. 7I) using ImageJ/Fiji (NIH). All ROIs were pooled for each of two images of the internal granule cell layer in non-unipolar brush cell expressing regions, and the pooled data from each confocal image (n=10) were analyzed per animal (N=5).

### Electrophysiology

To prepare acute brain slices for recording from cerebellar granule cells, brains from juvenile (6–8 weeks) or young adult (3–6 months) *shank3*^*Δex4−22*^ wildtype, heterozygous, and homozygous knockout mice were rapidly removed and placed in ice-cold sucrose slicing solution. This solution contained the following components (in mM): 2.5 KCl, 0.5 CaCl_2_, 4 MgCl_2_, 1.25 NaH_2_PO_4_, 24 NaHCO_3_, 25 glucose and 230 sucrose. The brain was then mounted to a holder and encased in agar and sliced parasagittally (250 μm) using a Compresstome VF-200 (Precisionary Instruments). The cerebellar slices were then transferred to a recovery solution that included the following components (in mM): 85 NaCl, 2.5 KCl, 0.5 CaCl_2_, 4 MgCl_2_, 1.25 NaH2PO_4_, 24 NaHCO_3_, 25 glucose and 75 sucrose, maintained at 32 °C [87]. After 30 min of recovery, cerebellar slices were transferred to room temperature artificial cerebral spinal fluid (ACSF) containing (in mM): 124 NaCl, 26 NaHCO_3_, 1 NaH_2_PO_4_, 2.5 KCl, 2 MgCl_2_, 10 D-glucose, 2.5 CaCl_2_. All solutions were saturated with 95% O2 and 5% CO2, had a pH of 7.3–7.4 and osmolarity of 300–310 mOsm. Slices were transferred to a custom recording chamber on an upright Olympus BX51WI microscope and cerebellar granule cells in the internal granule cell layer in lobules 4–5 were visualized with a 60X water-immersion objective using infrared differential interference contrast. ACSF was continuously perfused into the chamber at the rate of 3–5 ml/min maintained at 32–34 °C.

Whole-cell voltage-clamp recordings of visually identified CGCs were made using borosilicate patch pipettes (1.5mm OD/0.86mm ID) pulled with a P-1000 micropipette puller (Sutter Instruments) to have a tip resistance of (5–8 MΩ) when filled with CsCl-based internal solution (E_Cl_ = 0 mV) that contained (in mM): 130 CsCl, 4 NaCl, 0.5 CaCl_2_, 10 HEPES, 5 EGTA, 4 Mg-ATP, 0.5 Na-GTP, and 5 QX314 with pH adjusted to 7.2–7.3 with CsOH and an osmolarity of 280–290 mOsm [88,89]. Whole-cell patch-clamp recordings were acquired with a Multiclamp 700B amplifier (Molecular Devices) and sampled at 20 kHz (10 kHz low pass filter) with a Digidata 1440A (Molecular Devices). Following formation of a gigaseal (>1GΩ), the whole-cell configuration was produced by application of rapid negative pressure to the pipette. Whole-cell membrane properties were determined by applying a 10-mV hyperpolarizing voltage step from the initial holding potential (−60 mV) in voltage-clamp mode. Whole cell recordings from CGCs had a series resistance of 20±5 MΩ and recordings with variation in series resistance of greater than 20% over the course of the recording were discarded. To isolate spontaneous excitatory postsynaptic currents (sEPSCs), CGCs were voltage-clamped at −60 mV and the GABA_A_ receptor antagonist, gabazine (10 μM; Tocris Bioscience) was present in the ACSF. Inward transient sEPSCs with a fast rise and exponential decay were analyzed over a 3–5 min period with Easy Electrophysiology Software (v2.6.0) by first-pass automatic threshold detection followed by manual inspection of events. All events from each CGC were used to construct a cumulative distribution histogram (Fig. 8C–F) for amplitude (1 pA bin size) or inter-event interval (IEI, 100 ms bin size). Event amplitude and IEI were averaged for each cell to generate group averages and for statistical comparisons between genotypes (Fig. 8C–F inset). Individual sEPSC amplitude histograms (5 pA bin) were constructed for each CGC and normalized to the total number of events. To reduce the impact of CGCs with high sEPSC frequencies across recordings, these normalized histograms created for each CGC were then averaged across groups (Fig. 8G) and fit with a gaussian function (Fig. 8H).

### Statistical Analysis

Mice of all three *Shank3*^*Δex4−22*^ genotypes and both sexes at two separate age groups (juvenile and adult) were evaluated in all behavioral assays and in electrophysiology experiments with no mice evaluated at more than one age. Automated and manual determination of dependent variable values in EthoVision XT 17.5 were analyzed using SPSS 29 (IBM) and Igor Pro 8 (Wavemetrics). For comparison of group effects on dependent variables, a 3-way ANOVA (genotype, age, sex) or 3-way repeated measures ANOVA (MANOVA) were used for data with equal variance based on the median (Levene’s Test). Bonferroni correction for multiple comparison post-hoc tests on the estimated marginal means was used for pairwise comparisons to identify differences between genotypes with different ages and sexes when 3-way ANOVAs indicated significant main effects or interactions for those terms with genotype. For all behavioral assays, data are shown separated by genotype and age with additional separation of data by sex. When Mauchly’s test of sphericity was significant for MANOVAs, the Huynh-Feldt tests were used to determine time effects (open field, rotarod). For data without equal variance (Levene’s Test, *p* < 0.05), nonparametric Kruskal Wallis H tests were used to identify significant genotype effects within ages and within sexes at each age since SPSS does not allow for multiple independent variables to be included in Kruskal Wallis H test. Mean colocalization values from each confocal image were compared to determine SHANK3 expression differences between MF terminal types were with an independent samples t-test. A t-test was used for comparison of average synaptic event amplitudes, interevent intervals, and percentages of events within each 5 pA histogram bin between wildtype and knockout mice within age groups. All data values are reported as mean ± standard error (SEM) with individual markers representing the value for each individual observation, which is the animal (N) for all behavioral assays, the image (n) for confocal analysis, and the cell (n) for electrophysiology assays.

## Results

### Anxiety-like behavior increases with age in absence of shank3^Δex4–22^.

**Figure 1.**
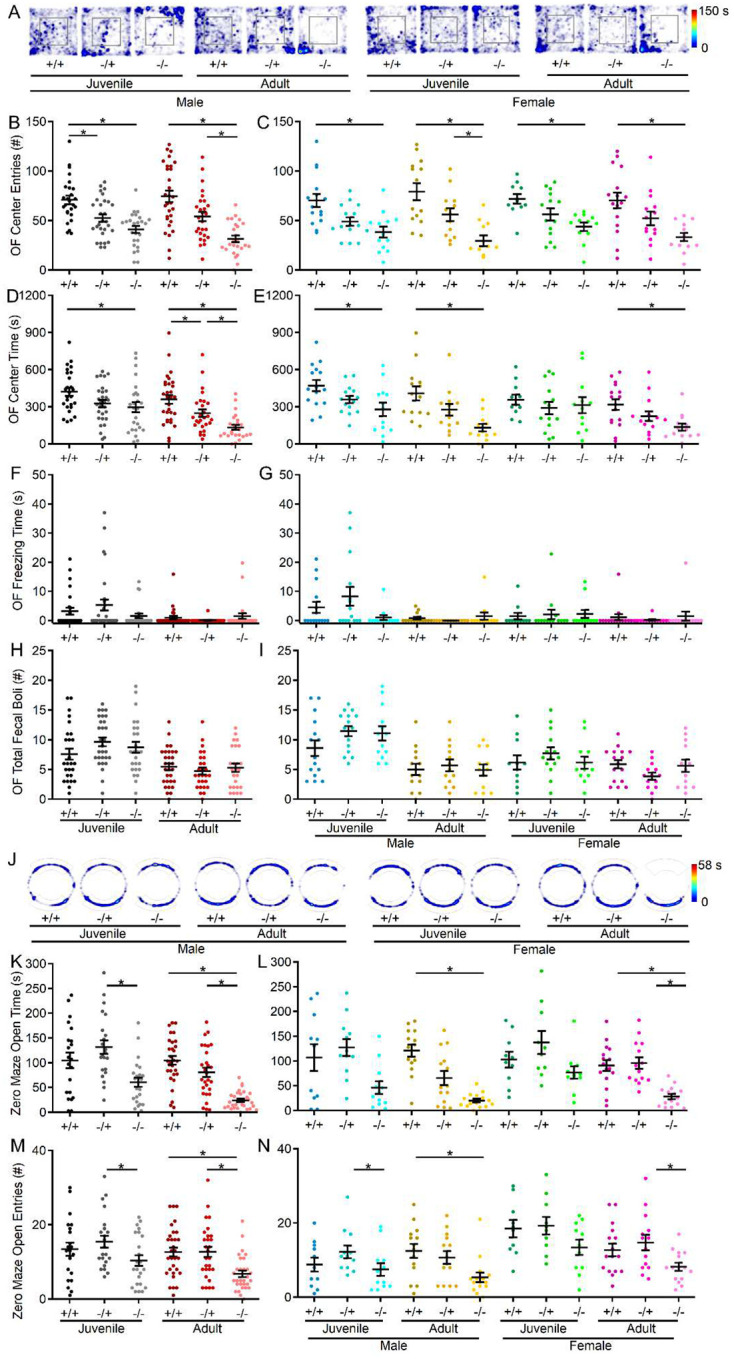
S*hank3*^*Δex4−22*^ KO mice display greater levels of anxiety with age. (**A**) Representative heatmaps of time spent in each area of the open field arena for one mouse of each genotype, age, and sex. (**B-I**) Individual (circles) and mean ± SEM (black bars) of the number of center entries (**B,C**), total open field center time (**D, E**), total freezing duration during open field exploration (**F**, **G**), and total number of fecal boli at the end of open field exploration (**H, I**) for each genotype at both ages (**B, D, F,** and **H**) and further separated by sex (**C, E, G,** and **I**). (**J**) Representative heatmaps of time spent in each area of the zero maze for one mouse of each genotype, age, and sex. (**K-N**) Individual animal (circles) and group mean ± SEM (black bars) of total open arm center time (**K, L**) and the number of open arm entries (**M, N**) in the elevated zero for each genotype at both ages (**K, M**) and further separated by sex (**L, N**). For **A**, and **J**, the color scale bar at right applies to all heatmaps in the corresponding assay. N = 22–30 mice/group for each genotype at each age and N = 10–16 mice/group for each sex within each genotype at each age. *p<0.05 for post-hoc test between genotypes with a Bonferroni correction.

To investigate anxiety-like behavior in *shank3*^*Δex4−22*^ mice, behavior in the open field and elevated zero maze tests were conducted. In the open field (Fig. 1A), *shank3*^*Δex4−22*^ knockout mice entered the center area less frequently at both ages compared to wildtype counterparts. This effect was observed in both males and females (Fig. 1B, C). Except for the juvenile knockout females, the time spent at the center of the open field was also reduced in all other *shank3*^*Δex4−22*^ knockout groups (Fig. 1D, E). No genotype effects were detected in total freezing duration (Fig. 1F, G) or in the number of fecal boli deposited (Fig. 1H, I) at the end of the session. Although a significant interaction between age and genotype was not detected in the open field center area measures, the elevated zero maze was used as an alternate more sensitive measure of anxiety-like behavior (Fig. 1J). In the zero maze, adult *shank3*^*Δex4*−*22*^ knockout mice spent less time in open arms (Fig. 1K, L) and entered open arms less often (Fig. 1M, N) compared to *shank3*^*Δex4−22*^ wildtype and heterozygous mice. There was also a significant interaction between age and genotype corresponding to an absence of significant difference between zero maze open arm time between *shank3*^*Δex4−22*^ wildtype and knockout juvenile mice. The increased avoidance of the open/exposed areas in both assays is indicative of heightened anxiety-like behavior with reduced s*hank3*^*Δex4−22*^ expression that escalates during the juvenile to adult transition.

### Reduced locomotor activity in shank3^Δex4−22^ knockout mice is consistent throughout early maturity.

**Figure 2.**
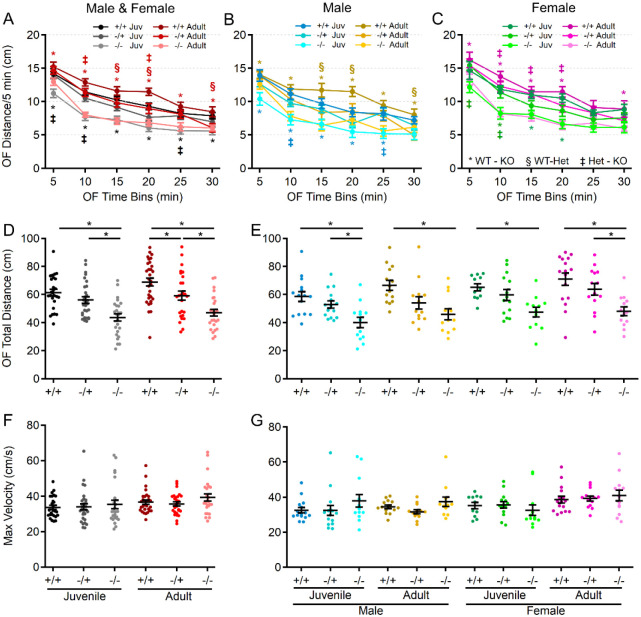
Juvenile and adult s*hank3*^*Δex4−22*^ KO mice display reduced exploratory and locomotion behavior. (**A-C**) Mean ± SEM of the total distance moved within each 5 min period of open field exploration for each genotype at both ages (**A**) and further separated into males (**B**) and females (**C**) at each age. (**D-G**) Individual animal (circles) and group mean ± SEM (black bars) of the total cumulative distance moved (**D, E**) and maximal linear movement velocity detected (**F, G**) during open field exploration for each genotype at both ages (D, F) and further separated by sex (**E, G**). N = 22–30 mice/group for each genotype at each age and N = 10–16 mice/group for each sex within each genotype at each age. In open field distance time plots (**A-C**), symbols correspond to *p*<0.05 in post-hoc comparison of genotypes within age and/or sex: * WT-KO, ‡ Het-KO, and § WT-Het, while *p<0.05 in scatter dot-mean plots (**D-G**) in post-hoc comparisons between genotypes, all with a Bonferroni correction.

Spontaneous locomotion in the open field was evaluated as an indicator of gross motor ability, exploratory behavior, and basal spontaneous activity (Fig. 2). Analysis revealed main effects of genotype, age, and sex, but no significant interactions among these variables. At both ages and in both sexes, s*hank3*^*Δex4−22*^ knockout mice demonstrated reduced spontaneous locomotion relative to wildtype mice (Fig. 2A–E), which persisted throughout the 30 min session (Fig. 2A–C). Despite the lack of significant interaction between genotype and age, the difference in total distance moved in the open field between s*hank3*^*Δex4−22*^ wildtype and knockout mice was greater in the adult group compared to the juvenile group. There was a similar age-dependent shift in the adult heterozygous s*hank3*^*Δex4−22*^ mice which also significantly differed from wildtype mice in total distance moved (Fig. 2D).

### Motor performance declines with age in the absence of shank3^ex4–22^.

**Figure 3.**
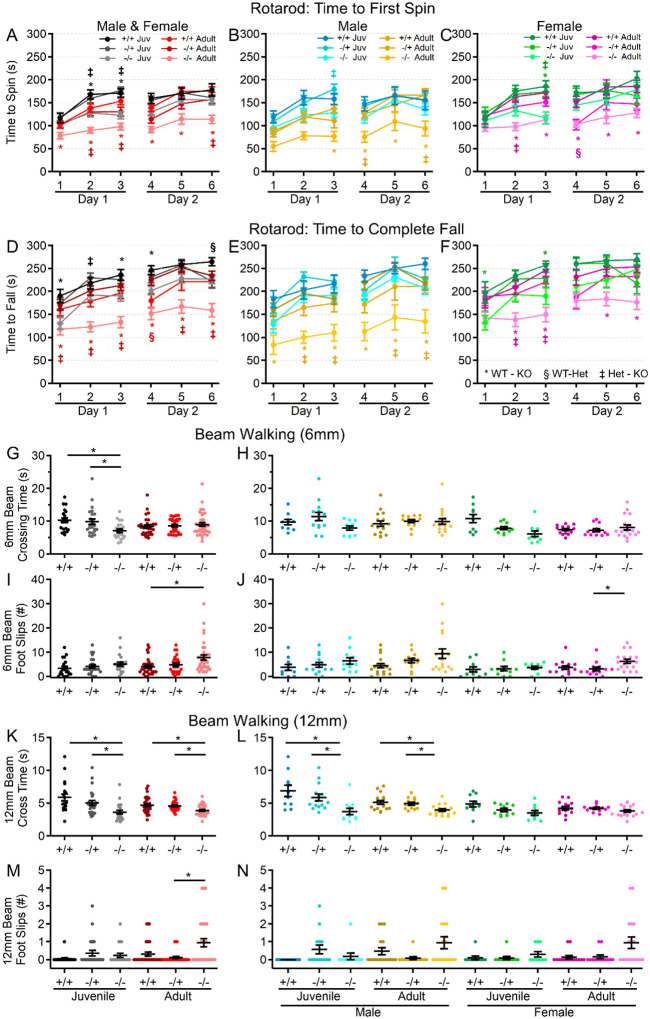
S*hank3*^*Δex4−22*^ KO mice develop motor function deficits with age. (**A-F**) Mean ± SEM of the time until the mouse rotates completely around the rotarod (**A-C**) or falls to the landing platform (**D-F**) for three subsequent accelerating rotarod tests (4–40 RPM, 5 min) repeated over two days total for each genotype at both ages (**A, D**) and further separated into males (**B, E**) and females (**C, F**) at each age. (**G-N**) Individual (circles) and mean ± SEM (black bars) of the time to cross (**G, H, K,** and **L**) and the number left and right total foot slips (**I, J, M,** and **N**) on a 6mm wide (**G-J**) and 12mm wide (**K-N**) beam for each genotype at both ages (**G, I, K,** and **M**) and further separated by sex (**H, J, L,** and **N**). N = 20–35 mice/group for each genotype at each age and N = 10–18 mice/group for each sex within each genotype at each age. In open field distance time plots (**A-F**), symbols correspond to *p*<0.05 in post-hoc comparison of genotypes within age and/or sex: * WT-KO, ‡ Het-KO, and § WT-Het, while *p<0.05 in scatter dot-mean plots (**G-N**) in post-hoc comparisons between genotypes, all with a Bonferroni correction.

**Figure 4.**
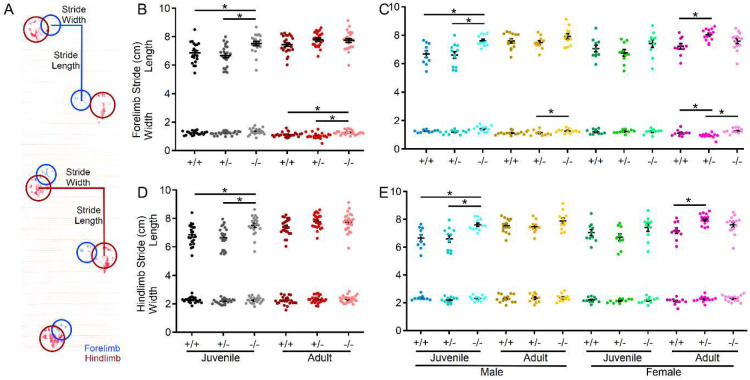
S*hank3*^*Δex4−22*^ KO mice develop an elongated stride length as juveniles. (**A**) Sample gait analysis raw data with location, stride length, and width of the forelimb identified in blue and hindlimb in red. (**B-E**) Individual (circles) and mean ± SEM (black bars) of the forelimb stride length and width (**B,C**) and the hindlimb stride length and width (**D, E**) for each genotype at both ages (**B, D**) and further separated by sex (**C, E**). N = 22–25 mice/group for each genotype at each age and N = 10–14 mice/group for each sex within each genotype at each age. *p<0.05 for post-hoc test between genotypes with a Bonferroni correction.

Since motor performance is often affected in ASD and is one area in which PMS patients experience regression, motor performance of s*hank3*^*Δex4−22*^ mice was assessed in multiple assays, including the rotarod, beam walking, and gait analysis. As an indicator of gross motor ability, maximal linear velocity throughout the entire 30 min open field session was assessed in all groups, but there was no difference due to genotype (Fig. 2F, G), but there was a significant main effect of age and sex. To provide a comprehensive view of motor and vestibular ability using the accelerating rotarod, the time (corresponding to rotation speed) an animal attached to the rod for one complete revolution (Fig. 3A–C) and the time when they completely fell off the rotarod to the landing platform (Fig. 3D–F) were both recorded. Latency to first spin times were shorter than the latency to fall times and juvenile mice generally had longer latencies than adult mice as did female mice relative to male mice at either age. Although both measures of rotarod performance identified main effect of genotype, age, and sex, as well as significant age and sex interactions in later trials, the time to fall measure more robustly detected significant interactions between genotype and age (Fig. 3D–F) on rotarod performance. Specifically, adult male and female *shank3*^*Δex4−22*^ knockout mice performed worse than wildtype and heterozygous mice on most trials. However, these deficits were only beginning to emerge in juvenile knockout mice at some trials (Fig. 3D–F). In assessing the ability to reliably traverse a narrow beam (6 mm or 12 mm wide) as an additional assessment of motor coordination and balance (Fig. 3G–N), juvenile and adult female s*hank3*^*Δex4−22*^ knockout and heterozygous mice displayed a reduce time to travel the beam to a closed goal box relative to wildtype mice (Fig. 3 G, H, K, L). However, only adult s*hank3*^*Δex4−22*^ knockout mice displayed an increased number of foot slips when traversing the 6 mm (Fig. 4I, J) and 12 mm wide beams (Fig. 3M, N), which is in line with similar age-specific deficits in rotarod performance. As a final assessment of motor function, gait analysis (Fig. 4A) was performed to assess changes in forelimb and hindlimb stride length and width (Fig. 4). With the exception of hindlimb stride width, there were main effects of genotype and age on the remaining three parameters (Fig. 4). Specifically, there was a significant elongation of the forelimb and hindlimb stride length in juvenile s*hank3*^*Δex4−22*^ knockout mice, which appeared to be more prominent in male mice. A similar trend was present in adult mice but may have been reduced due to variation in animal size by that age (Fig. 4B–E). These data support the idea that brain areas necessary for balance and motor coordination, including the striatum and cerebellum that both express high levels of *shank3*, may undergo escalating levels of disruption during early adulthood with the absence of s*hank3*^*ex4–22*^.

### Increased repetitive self-grooming behavior in male shank3^Δex4−22^ knockout mice.

**Figure 5.**
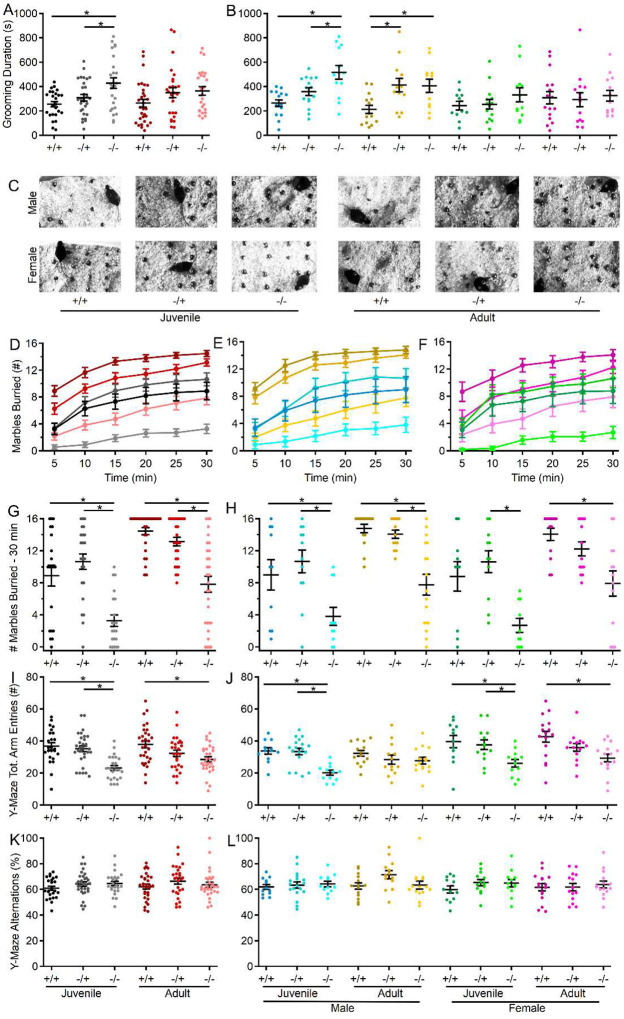
Loss of s*hank3*^*ex4−22*^ increases repetitive behavior and decreases exploratory behavior sex- and age-dependently. (**A, B**) Individual (circles) and mean ± SEM (black bars) of the total duration of grooming time during open field exploration for each genotype at both ages (**A**) and further separated by sex (**B**). (**C**) Representative images of marble location after 30 min in the marble burying arena for one mouse of each genotype, age, and sex. (**D-F**) Mean ± SEM of the number of marbles buried after each 5 min period during the marble burying assay for each genotype at both ages (**D**) and further separated into males (**E**) and females (**F**) at each age. (**B, H**) Individual (circles) and mean ± SEM (black bars) of the number of marbles buried after 30 min for each genotype at both ages (**G**) and further separated by sex (**H**). (**I-L**) Individual animal (circles) and group mean ± SEM (black bars) of the total number of arm entries (**I, J**) and percent of alternations (**K, L**) in the Y-maze for each genotype at both ages (**I, K**) and further separated by sex (**J, L**). N = 20–31 mice/group for each genotype at each age and N = 10–18 mice/group for each sex within each genotype at each age*p<0.05 for post-hoc test between genotypes with a Bonferroni correction.

To assess repetitive and exploratory behaviors in the absence of s*hank3*^*ex4−22*^, self-grooming activity in the open field and marble burying activity were assessed in all groups. Unlike other behaviors tested, juvenile mice lacking both copies s*hank3*^*ex4−22*^ displayed the most robust increase in time spent performing self-grooming behavior (Fig. 5A, B), with s*hank3*^*Δex4−22*^ knockout mice demonstrating significantly elevated grooming duration relative to heterozygous and wildtype mice (Fig. 5A). Notably, this increase in repetitive self-grooming appeared to be restricted to male mice with a significant interaction between genotype and sex identified (Fig. 5B).

### Reduced marble burying and exploratory behavior is pronounced in juvenile shank3^Δex4−22^ knockout mice.

In the marble burying assay (Fig. 5C), considered to identify anxiety-like, repetitive, or even exploratory behaviors, juvenile s*hank3*^*Δex4−22*^ knockout mice also demonstrated the most robust reduction in marbles buried (Fig. 5C, D, G), which persisted into adulthood. Although s*hank3*^*Δex4−22*^ knockout mice in all ages and sexes displayed reduced marble burying behavior, the effects were similar across ages and sexes (Fig. 5E, F, H). Given the increase in repetitive self-grooming behavior (Fig. 5A, B) in the absence of s*hank3*^*ex4−22*^, the decrease in marble burying behavior (Fig. 5C–H) may be more in line with elevated anxiety-like (Fig. 1) or reduced exploratory behavior (Fig. 2).

### Spatial working memory is not disrupted by the absence of shank3^ex4–22^.

Spatial working memory was evaluated through spontaneous exploration of the Y-maze. Although the total number of arm explorations was reduced in mice lacking s*hank3*^*ex4−22*^ (Fig. 5I, J), there was no difference in the percentage of those explorations that were novel alternations (Fig. 5K, L). The reduction in total number of arm entries observed in s*hank3*^*Δex4−22*^ knockout mice is consistent with reduced exploratory behavior observed in the open field (Fig. 2) and marble burying assay (Fig. 5C–H) for these same mice. However, the absence of any genotype effect on the percent of alternations (Fig. 5K–L), regardless of age or sex, suggests that spatial working memory is intact in the absence of s*hank3*^*ex4–22*^. *Mice lacking* s*hank3*^*ex4−22*^ *display reduced social preference, but not social novelty*.

**Figure 6.**
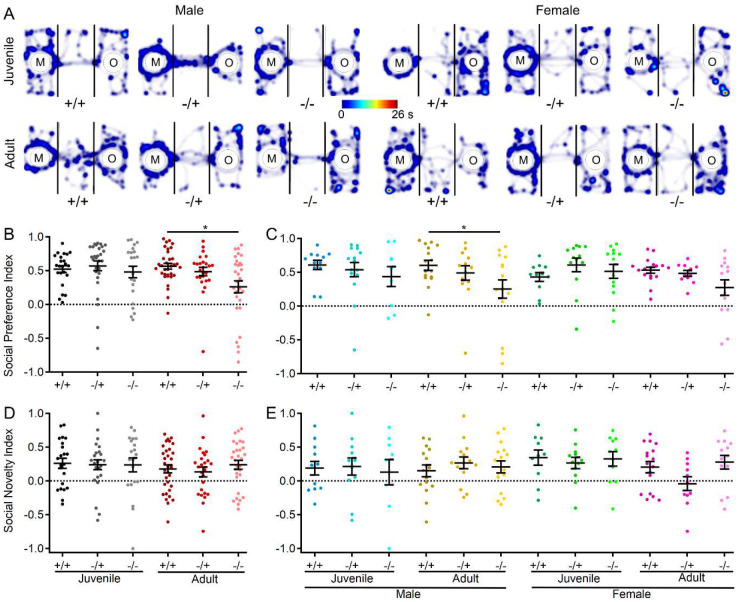
Male s*hank3*^*Δex4−22*^ KO mice develop reduced social preference with age in the three-chamber sociability assay. (**A**) Representative heatmaps of time spent in each area of the three-chamber arena for one mouse of each genotype, age, and sex. The color scale bar at center applies to all heatmaps in the **A**. (**B-E**) Individual (circles) and mean ± SEM (black bars) of the social preference index (**B, C**) and social novelty index (**D, E**) for each genotype at both ages (**B, D**) and further separated by sex (**C, E**). N = 23–33 mice/group for each genotype at each age and N = 9–17 mice/group for each sex within each genotype at each age. *p<0.05 for post-hoc test between genotypes with a Bonferroni correction.

In a final assessment of behavior in s*hank3*^*Δex4−22*^ mouse, a three-chamber sociability assay was conducted to evaluate both social preference (object vs. mouse) and social novelty preference (familiar mouse vs. novel mouse) [85]. In comparing the social preference index across groups, there was a general preference for social stimuli over non-social stimuli (Fig. 6A–C) and for novel over familiar mouse stimuli (Fig. 6D, E) across all three genotypes, both sexes, and age groups. Social preference index values displayed wide variability, with a significant main effect of genotype observed. Specifically, a significant reduction in the social preference index was identified in adult s*hank3*^*Δex4−22*^ knockout mice relative to wildtype mice (Fig. 6B). Although there were no main effects or interactions involving age or sex, data were further separated by sex for consistency. A significant reduction in social preference index was found only in adult male knockout mice (Fig 6C). In contrast, there were no genotype effects on preference for a novel target mouse over a familiar target mouse in the social novelty phase of the assay (Fig. 6D, E).

### Shank3 is present surrounding all cerebellar cortical mossy fiber terminals.

**Figure 7.**
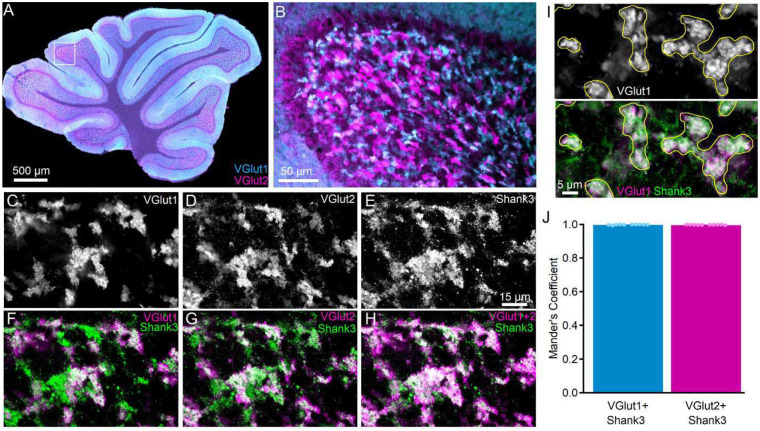
SHANK3 is expressed at CGC dendrites around VGlut1- and VGlut2-positive mossy fibers (MF) terminals. (**A, B**) Confocal fluorescence images at 4x (**A**) and 20x (**B**) demonstrating expression of VGlut1 (cyan) and VGlut2 (magenta) throughout the internal granule cell layer at mossy fiber terminals. (**C-H**) Grayscale (**C-E**) and pseudocolor (**F-H**) 60x confocal fluorescence single plane images of the same image location in the internal granule cell layer in parasagittal sections labeled with VGlut1, VGlut2, and SHANK3. Fluorescence color is assigned to enhance contrast in comparing magenta and green. (**F-H**) SHANK3 is expressed around VGlut1- and VGlut2-expressing terminals. (**I)** Example of how VGlut1-positive (white, top) terminals were used to define ROIs (yellow) for SHANK3 colocalization analysis. (**J**) Individual (circles) and mean ± SEM (bars) Mander’s coefficient for each analyzed image reflect similar colocalization of SHANK3 at VGlut1- and VGlut2-expressing mossy fibers. VGlut1-expressing (281 terminals) and VGlut2-expressing (285 terminals) were evaluated from n = 10 images per mossy fiber marker from N = 5 C57bl/6J mice.

The *shank3* gene is expressed in specific brain regions, including the striatum, cortex, hippocampus, thalamus, and cerebellum among some others [13,33]. However, despite involvement of the cerebellum in multiple motor and non-motor processes, little is known about the distribution and function of *shank3*/SHANK3 in the cerebellum other than its expression in CGCs [33,44–46,48]. To understand where SHANK3 is expressed in CGCs, parasagittal sections of wildtype C57bl/6J mice were immunostained for SHANK3 and markers of the two major classes of cerebellar cortical mossy fiber terminals (VGlut1 and VGlut2) that provide the primary input to CGCs (Fig. 7). Since VGlut1- and VGlut2-expressing MFs may arise from different sources [90], it was not surprising that their staining rarely overlapped within the internal granule cell layer (Fig. 7A, B). However, at nearly all MFs, regardless of whether they were of the VGlut1 or VGlut2 type, SHANK3 was expressed surrounding each terminal type (Fig. 7 C–H), indicating the broad presence of SHANK3 at all inputs to CGCs. Quantitative colocalization analysis of SHANK3 with either VGlut1 or VGlut2 supported this observation and that there was no significant difference (*t* (18) = 1.376, *p* = 0.186) in the colocalization of SHANK3 with VGlut1 (Mander’s Coefficient = 0.9986 ± 0.0005; 281 ROIs from n = 10 images from N = 5 mice) or VGlut2 and (Mander’s Coefficient = 0.9977 ± 0.0004, 285 ROIs from n = 10 images from N = 5 mice).

### Spontaneous excitatory synaptic events are larger in the absence of shank3^ex4–22^.

**Figure 8.**
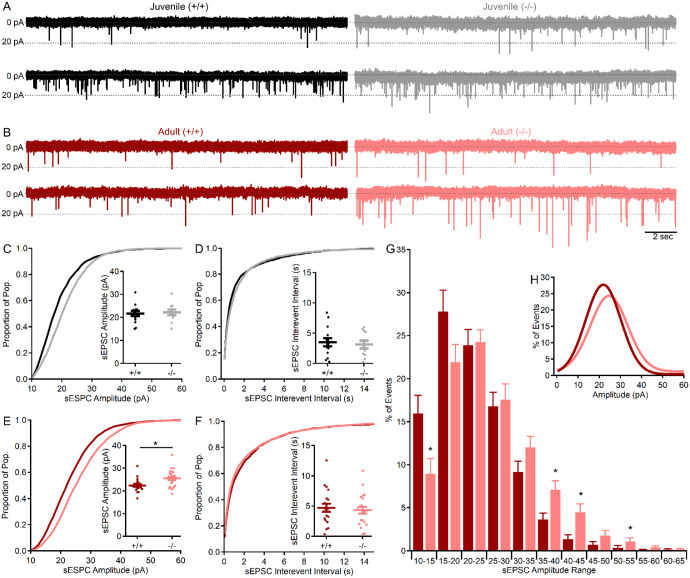
MF–CGC sEPSC amplitude is augmented in adult mice lacking s*hank3*^*ex4–22*^. (**A, B**) Representative (20 sec) traces of CGC sEPSCs (in 10μM gabazine) recorded from two genotypes each juvenile (**A,** black, gray) and adult (**B**, red, light red) wildtype (+/+) and knockout (−/−) s*hank3*^*Δex4−22*^ mice. Each trace is from a different CGC. (**C-F**) Cumulative distribution histograms for all events for each group with corresponding inset individual (circles) and mean ± SEM (bars) for sEPSC amplitudes (**C, E**) and interevent intervals (**D, F**) from juvenile (**C, D**) and adult (**E, F**) wildtype (+/+) and knockout (−/−) s*hank3*^*Δex4−22*^ mice. (**G**) Average normalized (to total event number) distribution histogram for sEPSC values from each CGC with the gaussian fit of the averaged distribution provided in the inset (**H**). n = 19 – 22 cells/genotype from N = 11 – 14 adult mice and n = 11 – 14 cells/genotype from N = 6 – 9 juvenile mice. For comparison of mean group sEPSC values (**C-F** insets) or averaged sEPSC histogram bin percentages (**G**) *p<0.05 for t-test between genotypes.

With the expression of SHANK3 in CGC dendrites encasing all glutamatergic MF terminals (Fig. 7), spontaneous non-NMDA receptor-mediated excitatory synaptic currents (sEPSCs) were evaluated in all wildtype and knockout *shank3*^*Δex4−22*^ mice at both ages to identify a relationship between behavioral phenotype and cerebellar glutamatergic CGC-MF function. Pharmacologically isolated sEPSCs (10 μM gabazine) recorded from CGCs (Fig. 8A; n = 1730 events in 14 cells from N = 9 wildtype mice and n = 1845 events in 11 cells from N = 6 knockout) of juvenile mice were comparable in both amplitude (Fig. 8C; *t*(23) = −0.29, *p* = 0.77) and frequency (Fig. 8D; t(23) = 0.39, *p* = 0.70). However, sEPSCs from adult CGCs (Fig. 8B; n = 2122 events in 19 cells from N = 11 wildtype mice and n = 2694 events in 22 cells from 14 knockout mice) were significantly larger in s*hank3*^*Δex4−22*^ knockout mice (Fig. 8E; *t*(39) = −2.82, *p* = 0.008), but occurred at similar frequencies in both genotypes (Fig. 8F; *t*(39) = 0.84, *p* = 0.40). The increase in the mean sEPSC amplitude averaged per cell (Fig. 8E inset) can also be observed in the rightward shift in the cumulative amplitude distribution histogram for all sEPSC amplitudes (Fig. 8E), with a similar trend observed in the amplitude distribution from juvenile animals (Fig. 8C).

Because the sEPSCs may be action potential-dependent or -independent and, as a result, single or multi-quantal, the distribution of sEPSC amplitudes was evaluated further (Fig. 8G, H). In order to reduce the impact of individual cells with high or low numbers of sEPSC events, an individual event amplitude histogram (5 pA bin) was constructed for each CGC and normalized to that neuron’s total number of events, with the resulting normalized histogram averaged across groups (Fig. 8G, H). The averaged histograms have a single peak (Fig. 8H) which was shifted from 15–20 pA in CGCs from wildtype mice to 20–25 pA in knockout mice, indicating that most events were due to release of single quanta and that the distribution of events was indeed shifted by ~5 pA or 25%. This progressive augmentation of non-NMDA receptor-mediated sEPSC amplitude in adult mice with the absence of s*hank3*^*ex4−22*^ suggests that there may be a change in the type or level of postsynaptic CGC AMPA or kainite receptors at the CGC-MF synapse. The absence of any sEPSC frequency changes and the single peak amplitude distribution suggests that presynaptic release is unlikely to be affected, which is in line with a lack of SHANK3 expression in MF terminals.

## Discussion

In an effort to understand how specific behaviors develop or regress in early adulthood in PMS and some forms of ASD, we evaluated the behavior of s*hank3*^*Δex4−22*^ mice [47], a pre-clinical animal model of PMS and ASD, at two specific time points (5–6 weeks and 3–5 months). Since these mice lack major *shank3* gene exons and all corresponding isoforms of the SHANK3 protein, the broad role of *shank3*/SHANK3 in neuronal and circuit function can be assessed in this mouse model. Evaluation of wildtype, heterozygous, and knockout mice on behavioral assays across a range of domains, including anxiety-like, motor, exploratory, memory, and social behavior, revealed that behavioral changes in the absence of s*hank3*^*ex4−22*^ fell into three categories. First, exploratory behavior (Fig. 2A–E, 5C–J), gait differences (Fig. 4), and repetitive behavior (Fig. 5A–H) were relatively well-established in juvenile mice, and little change was observed in these areas in adult cohorts. In contrast, loss of *shank3*^*ex4−22*^ led to increased anxiety-like behavior (Fig. 1A–E & J–N), disruption of motor coordination (Fig. 3), and reduced social preference (Fig. 6) that became more pronounced in adult mice relative to juvenile mice. Finally, s*hank3*^*ex4−22*^ loss did not seem to impact freezing (Fig. 1F, G), fecal boli deposits (Fig. 1H, I), gross motor ability (e.g. locomotion speed; Fig. 2F, G), or short-term spatial memory (Fig. 5K, L).

With this broad assessment of how behavioral phenotype develops or regresses in the absence of s*hank3*^*ex4−22*^ we also sought to identify a neural mechanism that may contribute to this regression. Based on the relatively late-stage development of the cerebellum [91] and the cerebellum’s interaction with multiple other brain areas, we hypothesized that the absence of *shank3* from this circuit may be particularly consequential. Immunohistochemical assessment of SHANK3 distribution revealed that it is present in CGC dendrites that encase nearly all glutamatergic MF inputs into the granule cell layer (Fig. 7) – a key site of signal integration in the cerebellum. A simple electrophysiological interrogation of this synapse determined that not only an enhanced MF-CGC synaptic response (Fig. 8), but also that this augmentation was specific to adult s*hank3*^*Δex4−22*^ mice, correlating with the development of anxiety-like behavior, disrupted motor, and social behaviors. The sEPSCs analyzed here to evaluate MF-CGC functional changes in the absence of *shank3* may represent pre- and postsynaptic effects in principle. However, given that the expression of *shank3/*SHANK3 is limited to CGCs at this synapse and that the sEPSC amplitudes reported are within the range of quantal event mEPSCs using similar configurations [92], we predict that the synaptic augmentation is likely due to the expression level or subunit composition of non-NMDA receptors at the MF-CGC synapse.

The behavioral data presented here, especially for the adult timepoint, are largely in line with previous studies conducted on the s*hank3*^*Δex4−22*^ mouse model at comparable ages of 3–10 months [46,47]. Of the behaviors assessed here and in prior assessments of the same [47] and similar [46] s*hank3*^*Δex4−22*^ mice, genotype-dependent differences in adult mice were most closely aligned in open field locomotion, rotarod, beam balance, and gait. However, the magnitude of some genotype-dependent differences in behavior for adult mice was somewhat smaller in this study than in previous reports, which may be due to some methodological differences. First, the background strain of the mice used here was a mixed C57bl/6NJ as provided by the vendor, while others used s*hank3*^*Δex4−22*^ mice on a C57bl/6Tac [47] or C57bl/6J [46]background. Perhaps more importantly, the behavioral data collected in this study occurred during each animal’s active dark cycle and in low light conditions, rather than during the inactive light cycle as done by others [46,47] that may introduce additional circadian-related variables. The current study was also sufficiently powered to detect sex effects, which allowed for the identification of significant genotype-sex interactions that identified increased stereotyped grooming and reduced social preference behavior in male s*hank3*^*Δex4−22*^ knockout mice. It is expected that the distribution of sexes, circadian effects, or both may impact the genotype-dependent changes in grooming and social behavior observed here relative to others [46,47]

Behavioral analysis has been conducted on several mouse lines lacking specific *shank3* exons and corresponding specific SHANK3 protein isoforms due to the distribution of promoter regions throughout the *shank3* gene. While the behavioral phenotyping work across the different *shank3* mutant mouse lines has largely been in a single age cohort or in neonatal or mature adult mice, much of this work has been summarized elsewhere, and the role of isoforms is discussed in detail below [47]. However, more recently some groups have specifically compared the behavioral phenotypes of the *shank3*^*Δex11*^[26,27], *shank3*^*Δex21*^[28], and the *shank3*^*Δex4−22*^ [29] across age groups (Table 1). While the methodological details of each of these studies differ from one another with respect to age ranges, all performed during the inactive light phase or did not clarify the timing, and some retested the same cohort of mice. Three of these multi-timepoint studies [26–28] evaluated behavior in mouse lines that only lack some SHANK3 isoforms, which may explain why only a limited subset of behaviors are evaluated and/or found to be different across genotypes regardless of age. Unlike prior longitudinal studies [26–29], where the same animals were subjected to the same behavioral experiments at different ages, potentially influencing the behavioral results at older ages due to task familiarization and learning ability, our findings based on separate age groups at two different time points provide a novel and more robust characterization of the behavioral changes between juvenile and adult knockout mice. Since the fourth study [29] evaluated a motor, social, exploratory, and anxiety-like behavior in the *shank3*^*Δex4−22*^ mouse at earlier developmental timepoints (2–8 weeks), the current study may combine with the work of Contestabile et al., to provide a more comprehensive understanding of how behavioral domains are affected by the loss of all SHANK3 isoforms over time.

Interpretation of behavioral phenotype data requires an understanding of the cellular-level impacts of each genetic manipulation, which is particularly important in the case of the different *shank3* deletion strains since each exon deleted will lead to loss of alternatively spliced specific SHANK3 isoforms [33,44–47]. Since each SHANK3 isoform is preferentially expressed at different levels across discrete brain areas [33], the behavioral impact of deleting a subset of exons and related isoforms depends on the role each isoform plays in different brain areas at specific times. For example, *shank3*-mutant mice missing exons 4–9 that only lack isoforms A-B (ankyrin-containing; [35,36,42–45] more commonly display stereotyped grooming behavior and altered social interactions. In contrast, mice with mutations affecting exons 11–22 that lack expression of isoforms C-D (non-ankyrin containing; [34,46,47,93,94]) more often display heightened avoidance behaviors, anxiety, deficits in sensory processing, and poor performance on cerebellar-dependent motor tasks [46,47]. Variation in isoform expression over time is also a key consideration in addition to the location of isoform expression. In an evaluation of the changes of isoform expression over time, SHANK3A and E peak in early development around 4 weeks of age and then decline, while SHANK3B levels remain relatively constant over time, but SHANK3C and D steadily increase during early development and remain high throughout adulthood in mice [33]. With the need to address all isoforms and account for key temporal progressions of isoform expression, the present study fills an important gap in the current literature.

Unfortunately, conditional knockout approaches deleting s*hank3*^*Δex4−22*^ from specific populations has only revealed that *shank3* in striatal or forebrain neurons account for changes in exploratory and repetitive self-grooming behavior [41] With the range of behavioral domains affected in mice with germline *shank3* exon deletions, the brain regions most disrupted by the loss of s*hank3*^*Δex4−22*^ to affect these other behaviors remain to be identified. The integration of timelines for behavioral phenotype development and isoform expression along with knowledge of brain regional variation in isoform expression perhaps guide future work aimed at addressing this issue. The behavioral regression observed in both humans [20–25] and mice (Table 1, this study) occurs over a time when isoform C and D expression is increasing to its steady state peak during adolescence and early adulthood [33]. This timeframe also aligns with final stages in the development of the cerebellar cortex, where *shank3* is expressed exclusively in cerebellar granule cells as isoforms C and D [44,65,95–97]. Guided by these data, our findings not only demonstrate the ubiquitous presence of SHANK3 at all MF-CGC synapses (Fig. 7), key sites for sensorimotor integration within the cerebellar circuit, but also reveals *shank3*-dependent changes in the function of this synapse (Fig. 8) that align with the timing of behavioral phenotype regression across juvenile and early adulthood. This novel finding of disrupted glutamatergic synaptic function in the absence of *shank3* provides evidence for cerebellar disruption in this animal model of PMS and ASD, while also highlighting the need to determine the specific cause of this synaptic augmentation (subunit change, receptor density increase, etc.).

Determining the *shank3*-brain region interactions that drive *shank3*-dependent changes in anxiety, motor function, social interactions, communication, and some forms of learning is crucial for understanding and treating PMS, and possibly ASD. The temporal relationship between cerebellar synaptic functional changes and behavioral regression in s*hank3*^*Δex4−22*^ mice suggests cerebellar involvement. Recent studies [21,23] have reported a significant correlation between age and the prevalence and severity of regression in cases of PMS. Interestingly, regression was primarily observed to impact fine and gross motor function and language skills, which are largely considered to be under cerebellar control. Although these symptoms typically begin at a young age, they tend to worsen during adolescence. Multiple studies indicate that pathophysiology of the cerebellum, likely via these non-motor connections, may actually be involved in the etiology of ASD [10,57–64]. Changes in cerebellar cortical size [98,99], development [100–102], and Purkinje cell (PC, principal cortical efferent) density [103,104] have all been linked to ASD diagnoses. In addition, cerebellar damage at birth [57,105] and dysfunction in known cerebellar-dependent behaviors or differences in cerebellar activity (e.g. fMRI) are also linked to ASD [106–112]

Perhaps due to its well-known role in motor learning and function, a role for cerebellar dysfunction across the broad range of ASD-like behaviors has been largely overlooked. However, it is clear that the cerebellum modulates non-motor circuits related to cognitive, social, emotional, and attention processes [68–71]. Clinical and cognitive data also indicate that cerebellar-specific dysfunction/damage leads to a spectrum of abnormal behaviors and processes (including motor dysfunction) akin to behavioral changes observed in ASD [59,99,104]. In addition to the high level of *shank3* expression, CGCs and other cerebellar cortical neurons expresses higher levels of ASD-linked genes than other brain regions classically evaluated, like the striatum, cortex, and hippocampus [7,65,95,96,113]. The non-motor functions for the cerebellum, expression of *shank3* and other ASD-linked genes in the cerebellar cortex, and a causative role for cerebellar dysfunction in the heterogeneous behavioral phenotypes of ASD [57–63] together support the need to understand the degree of cerebellar involvement and mechanisms causing cerebellar dysfunction in ASD. In animal models, either deletion or mutation in ASD-linked genes (*Tsc1*, *Shank2, Mecp2*) confined to cerebellar cortex or specifically Purkinje cells often lead to disrupted motor, cerebellar-dependent, and social behaviors, coupled with altered synaptic physiology and cellular morphology [40,114–117]. Deletion of ASD-linked genes (*Chd8*, *Ib2*) encoding for regulators of gene expression/cell signaling specifically from CGCs alter CGC excitability and synaptic physiology [118,119], coupled with disruption of motor coordination when a subset of GCs in anterior motor areas of cerebellum are affected.

## Limitations

Despite the significant findings of this study, other behavioral assessments, including evaluations of muscle strength (e.g. grip strength), cognitive function (e.g. Barnes or Morris Water maze), and communication (e.g. ultrasonic vocalizations) were not conducted. These tests could provide comprehensive insights into an even broader phenotypic manifestations associated with loss of SHANK3 at different developmental time points and in both sexes. Future investigations incorporating these behavioral tests are essential to delineate the multifaceted impacts of SHANK3 on overall behavioral functions. Furthermore, the role of SHANK3 in the cerebellum remains under-explored. While this study focused on SHANK3’s influence on synaptic mechanisms at MF-CGC synapses, the specific pathways and cellular mechanisms through which SHANK3 modulates synaptic function in this context remain to be fully elucidated. Further research exploring structural and functional changes at MF-CGC synapses is necessary to uncover the precise modulatory effects of SHANK3.

## Conclusions

The behavioral data presented here provide a comprehensive view of behavioral differences based on *shank3* expression across multiple domains and in both sexes in mice. The assessment of these behaviors at juvenile and adult time points reveals domain-specific behavioral regression that aligns with analogous timepoints in humans. Together, these data offer a comprehensive understanding of *shank3*-dependent behavioral changes and their variation over time when assessed during an animal’s active (dark) phase. Finally, in an effort to identify functional changes corresponding to genotypic and developmental behavioral phonotypes, electrophysiology data identify an augmentation of the MF-CGC synapse that may impact cerebellar input integration and downstream modulation of multiple motor and non-motor circuits/processes.

## Figures and Tables

**Table 1.** Comparison of studies demonstrating behavioral changes in *shank3* mutant mouse models with age.

Publication		Ferhat et al., 2023	Bauer et al., 2023	Thabault et al., 2023	Contestabile et al., 2023	Kshetri et al.. Current Study
**Model**		Δ11	Δ11	Δ21	Δ4–22	Δ4–22
**Isoforms Deleted**		a,b,c	a,b,c	a,c,d,e,f	a,b,c,d,e,f	a,b,c,d,e,f
**Testinq Liqht Cycle**		Light	N/A	N/A	Light	Dark
**Aqes (weeks)**		12, 32, 52	4–9, 13–18	10, 20,40	2–8	5–7, 12–20
**Separate Aqe Cohorts**		Y/N	N	Y	N/A	Y
**Sexes**		M/F	M/F	M/F	M/F	M/F
**Motor Function**	Rotarod		↓		↓	↓
	Beam Balance					↓
	Gait					–
	Speed					–
	Strength		↓			
**Social**	3 Chamber or Free Social Preference	–	–		↓	↓
	3 Chamber Social Novelty					–
	Sociosexual	↑				
	Ultrasonic Vocalizations	–	–			
**Exploratory**	Locomotion	↓	↓		–	↓
	Marble Burying		↓			↓
**Anxiety-like**	Open Field Center Time		↓		↓	↓
	Zero/Plus Maze Open Arm Time	–			↓	↓
**Repetitive/Stereotyped**	Grooming	↑	↑	↑		↑
	Rearing			↑		
	Nestlet Shreddinq		↑			
**Memory**	Y-Maze	–				–
	Barnes/Star Maze	–	–			

Abbreviations: Y = Yes, N = No, N/A = Not available, M = Male, F = Female, ↑ = increase, ↓ = decrease, - = no change in tested behaviors due to reduced *shank* expression

## Data Availability

The datasets used and/or analyzed during the current study are available from the corresponding author on reasonable request with statistical analysis results included with this published article’s supplementary information files.
